# Providing Alcohol-Related Screening and Brief Interventions to Adolescents through Health Care Systems: Obstacles and Solutions

**DOI:** 10.1371/journal.pmed.1000214

**Published:** 2010-03-09

**Authors:** Duncan B. Clark, Howard B. Moss

**Affiliations:** 1 Western Psychiatric Institute and Clinic, University of Pittsburgh School of Medicine, Pittsburgh, Pennsylvania, United States of America; 2 National Institute on Alcohol Abuse and Alcoholism, National Institutes of Health, Bethesda, Maryland, United States of America

## Abstract

Duncan Clark and Howard Moss identify obstacles to alcohol-related screening and treatment for adolescents and propose policy solutions.

Adolescent binge drinking and related disorders are worldwide public health problems [1]. The European School Survey Project on Alcohol and Other Drugs [2] surveyed 16-year-old adolescents in 35 countries. Defined as the consumption of five or more drinks per episode, binge drinking had occurred in 43% of these adolescents in the prior 30 days. Eleven countries were noted to have rates of over 50% (e.g., United Kingdom 54%; Portugal 56%) and all but two countries had rates over 30%. In the US, about one-third of high school seniors reported binge drinking in the prior two weeks [3]. Comparable adolescent alcohol involvement has been noted for some Western Pacific countries, including Australia and New Zealand [1].

For over a decade, governmental and professional organizations such as the World Health Organization (WHO), the US Surgeon General, the American Medical Association, and the American Academy of Pediatrics have called on health care practitioners to become more involved in providing screening, brief intervention, and referral for treatment (SBIRT) for adolescent drinkers [4]–[8]. While often somewhat general, SBIRT guidance has been offered by some organizations ([4] – see also Box 1) and in review articles [9]. Moreover, although there is a consensus that the health care system is an appropriate SBIRT venue, most adolescents visiting health care providers do not receive these services [10]. This Policy Forum identifies the problems impeding SBIRT for adolescents and proposes some solutions.

**Box 1. The American Academy of Pediatrics [4] Recommendations**

- Evaluate alcohol use as a routine part of risk behavior assessment
- Recognize signs and symptoms of alcohol abuse
- Discuss the hazards of alcohol use
- Strongly advise patients against the use of alcohol
- Discourage parents from allowing underage drinking at home
- Refer patients for further assessment and treatment as indicated

## SBIRT Goals

Most SBIRT recommendations do not take into consideration developmental changes that occur during the course of adolescence. For example, the WHO recommendations to health care practitioners providing SBIRT to young people are intended to apply to patients aged 10 through 24 years [8]. Alcohol use and related problems increase dramatically from age 12 to age 20 [11]. In some health care settings, particularly those serving older adolescents, alcohol use disorders (AUD) occur at rates sufficient to justify the AUD focus that is standard for adults [12]. In early adolescence (ages 12 to 14 years), however, significant alcohol use is relatively unusual [13]. The identification of young adolescents with alcohol use, including those without an AUD diagnosis, would provide an opportunity for preventive action in high-risk patients [13]. In middle adolescence (ages 15 to 17 years), binge drinking emerges, with its potential for accidental death and injury [14]. In late adolescence (ages 18 to 20 years), AUD occurs at rates higher than in any adult age group [11].

### Proposal

In our opinion, age-related emphases on promoting alcohol abstinence and identifying binge drinkers would improve the developmental specificity and applicability of SBIRT approaches. Particularly in younger adolescents, alcohol abstinence should be the SBIRT goal. In middle adolescence, screening for binge drinking would provide a focus for prevention activities and would also identify most adolescents with an AUD. Exclusively focusing on AUD is appropriate for patients in late adolescence.

## Screening Methods

Several screening methods that target AUD have been developed for adolescents. Screening approaches recently studied include those based on alcohol use frequency [15], alcohol-related problems [16], or problems with alcohol and other drugs [17]. The available results suggest that each method may yield suboptimal sensitivity or specificity in some settings. The most studied screening instrument is the AUDIT. The AUDIT [18] is a ten-item screen including three consumption items and seven problem items with up to five response options for each item. The AUDIT has been translated into several languages, and has been studied in countries in North and South America, Europe, Asia, and the Western Pacific [18]. Adolescent studies of the AUDIT have yielded sensitivity rates ranging from 54% to 87%, specificity rates from 65% to 97%, and optimal scores ranging from 2 to 10 [18]. When directly compared, other methods have typically not been found to improve upon the AUDIT [16]. For example, CRAFFT is a 9-item questionnaire designed to screen for alcohol and other drug use disorders with acceptable psychometric properties in some studies [17] but poor specificity reported in some settings [16].

Very brief screening methods have been described with promising results. Using one question on the frequency of drinking episodes, a threshold of three or more episodes per month was found to identify adolescents with AUD at 90% sensitivity and 84% specificity [15]. Endorsing at least one of two DSM-IV AUD items (i.e., hazardous use and drinking more or longer than intended) identified adolescents with AUD at 88% sensitivity and 90% specificity [19]. These methods have not been otherwise tested.

### Proposal

Our view is that none of the available screening methods has been shown to have the full complement of characteristics that would warrant widespread use. In addition to inconsistent psychometric results, the AUDIT and CRAFFT multi-item questionnaires utilize administration and scoring procedures that may not readily integrate into typical clinical practices. Additional research is needed to develop and validate very brief screening methods that may be applied by interview or questionnaire methods and may thus be readily incorporated into current and future clinical practices. Furthermore, the exclusive focus on AUD may miss opportunities to identify at-risk adolescents. Screening methods for patients in early and middle adolescence should incorporate the assessment of alcohol use patterns to facilitate preventive interventions.

## Brief Interventions

Interventions for the prevention and initial treatment of AUD in adolescents have been tested in a variety of health care settings, including emergency departments and college health clinics [20],[21]. While alcohol-related brief interventions for adults have shown variable efficacy [22], the 2004 US Preventive Services Taskforce found “good evidence” and provided a “grade B recommendation” supporting SBIRT for adults [23]. By contrast, this group concluded that the evidence was “insufficient to recommend for or against screening and behavioral counseling interventions to prevent or reduce alcohol misuse by adolescents in primary care settings.” Similarly, the American Academy of Pediatrics [4] recommendations to pediatricians do not include suggestions for brief interventions “because the data for such management options…are not yet conclusive.”

Encouraging results have been recently reported with some brief interventions implemented in health care settings. An approach called motivational interviewing was compared to feedback only in 198 adolescents and young adults with problem alcohol use seen in a US emergency department [21]. Counselors providing motivational interviewing received 30 hours of training and weekly supervision. Provision of the motivational interviewing intervention required 30 to 45 minutes. Over a one-year follow-up period, those receiving motivational interviewing reported significantly less alcohol use. A prior study by these investigators [24] found a significant motivational interviewing effect on alcohol-related consequences but not on alcohol use. A Dutch study conducted in schools compared a similar motivational interviewing approach to an information-only control [25] and did not find a significant effect of motivational interviewing on subsequent alcohol use. An alternative approach minimizing practitioner training and office time relies on computer-administered interventions. In a study conducted in New Zealand [26], students visiting a university primary health care service received an information pamphlet, a single-session Web-based intervention, or a Web-based intervention with multiple sessions. Compared to the control intervention, the Web-based intervention groups reported less alcohol involvement through a one-year follow-up period. Adolescents' preference for computer-administered assessments [27] and the potential for anonymous participation in Web-based interventions may enhance the acceptability of such approaches for those with confidentiality concerns.

### Proposal

In our opinion, the extensive training and office intervention time required by motivational interviewing makes this approach unlikely to be adopted in primary care clinics or emergency departments. A consensus is needed to define the operating constraints in such settings that need to be considered in designing brief interventions. Web-based approaches may be more feasible, and additional research is needed to develop and test computer-assisted interventions for adolescents seen in health care settings.

## Referral for Treatment

Relatively few major studies have examined the effects of treatment approaches on alcohol consumption outcomes for adolescents with AUD [28]. Multisystemic therapy was developed as a comprehensive approach for adolescents involved in the juvenile justice system. Multisystemic therapy includes therapy sessions in the home, resources provided to parents, and interventions to address academic issues. In a study examining substance use outcomes [29], multisystemic therapy participants received 130 days of treatment with 40 hours of direct therapist contact. Compared to those receiving usual community services, adolescents receiving multisystemic therapy reported less alcohol, marijuana, and other drug use. In a study of four interventions for adolescents with substance problems [30], family therapy or family therapy with cognitive behavior therapy showed advantages over a group intervention or cognitive behavior therapy alone on marijuana use levels at a four-month follow-up. No significant differences among treatment groups were noted for alcohol use. In a study comparing cognitive behavior therapy and a psychoeducational intervention [31], adolescents in both conditions showed reduced alcohol use over a three-month period, but the effects of these interventions were not significantly different.

### Proposal

In our opinion, comprehensive treatment programs have been shown to be effective. The extent to which the less comprehensive treatment typically available is effective remains unclear. Even in the most affluent nations, there are shortages of specialized treatment programs and providers qualified to provide comprehensive treatment for adolescents with AUD [32]. Systematic information on local addiction treatment facilities capable of providing high-quality care to adolescents needs to be more widely available to facilitate rational treatment referral patterns. Unfortunately, detailed information about the availability, quality, effectiveness, and insurance coverage for local services will likely reveal system-wide limitations. Comprehensive adolescent addiction treatment needs to be more available.

## Confidentiality Concerns

Some adolescents have expressed apprehension that revealing their alcohol use may lead to conflicts with parents and other adverse consequences [33]. While both parents and adolescents are generally amenable to SBIRT [34], adolescents most in need of SBIRT are also the most likely to have confidentiality concerns [35]. Health care practitioners' assurance of confidentiality has been shown to improve adolescents' willingness to disclose this information [36]. In some cases, however, assurances of confidentiality may be at odds with state regulations, and some parents may expect that health care practitioners will disclose adolescent reports. In the US at least, adolescents are typically covered by parent insurance policies, and information provided on claims sent to parents may indirectly compromise confidential information. Perceptions of confidentiality may be enhanced by the collection of screening information using electronic devices, such as a personal digital assistant [37] or computer [27].

### Proposal

Clear practice policies regarding parental disclosure need to be established and communicated to parents and adolescents. Procedures for billing must ensure that confidential information is not inadvertently breached by billing codes and statements. The development and implementation of electronic data collection approaches may also improve adolescents' disclosure.

## Support from Health Care Agencies

In an evaluation of SBIRT for alcohol-related problems in 12 countries in Europe, Asia, and the Western Pacific, the WHO cites lack of governmental support and insufficient reimbursement as important obstacles to implementation [7]. In Australia, for example, SBIRT implementation by general practitioners was noted as being hampered by “logistical barriers, such as a lack of time and heavy workloads.” In Bulgaria, general practitioners were described as “overloaded.” Progress has been made toward providing resources for SBIRT in some countries. In the past two years, the US Centers for Medicare and Medicaid Services and the Federal Employee Health Benefits Program have begun supporting SBIRT with new billing codes for Commercial Insurance and the Health Care Services Procedures Coding System. The extent to which increased support for SBIRT improves implementation in health care settings will be important to determine.

### Proposal

In our opinion, insufficient support for alcohol-related SBIRT is evident in many countries. In the US, health insurance reimbursement rates and actual payments are typically insufficient, relative to the skills and time required, to fully support SBIRT practices. Fiscal and administrative support for SBIRT activities needs to reflect the resources required to provide these services.

## Conclusions

Health care practitioners have been exhorted to routinely provide SBIRT to underage drinkers by international, governmental, and professional organizations. Our evaluation of the status of these efforts indicates that substantial obstacles need to be overcome for this aspiration to be realized. In our view, these obstacles and their solutions are relevant in most of the countries facing significant problems with adolescent alcohol involvement. Our proposed solutions include a broader developmental perspective, the development of more effective prevention and intervention methods, and the provision of increased support for SBIRT services. SBIRT goals for adolescent patients need to be expanded from an exclusive focus on AUD to alcohol abstinence promotion and binge drinking prevention. Screening and assessment methods applicable in typical clinical practice settings need to be developed and validated. Brief interventions that are effective and feasible in typical health care settings need to be developed. While acknowledging that the interventions offered in the adolescent addiction treatment system are often imperfect, more comprehensive and systematic information about available local resources need to be accessible to health care practitioners. Confidentiality concerns need to be managed to facilitate adolescent participation. Adequate support for health care practitioners undertaking these challenging tasks will also be required. Despite these obstacles, many practitioners have been willing to provide SBIRT to their adolescent patients. Widespread routine implementation of SBIRT for underage drinkers in health care settings will occur when the clinical value of improved screening and brief intervention methods have been verified, adolescents may confidentially provide valid reports of their alcohol involvement without fear of reprisals, adolescent-focused addiction treatment services become more available and affordable, and health care practitioners are fairly compensated for these efforts.

## Abbreviations

AUD: alcohol use disorders
SBIRT: screening, brief intervention, and referral to treatment
